# Aerodynamic System Machine Learning Modeling with Gray Wolf Optimization Support Vector Regression and Instability Identification Strategy of Wavelet Singular Spectrum

**DOI:** 10.3390/biomimetics8020132

**Published:** 2023-03-23

**Authors:** Mingming Zhang, Pan Kong, Aiguo Xia, Wei Tuo, Yongzhao Lv, Shaohong Wang

**Affiliations:** 1 Faculty of Science, Beijing University of Technology, Beijing 100124, China; mmzhang@bjut.edu.cn; 2 Key Laboratory of Modern Measurement and Control Technology, Beijing Information Science and Technology University, Beijing 100192, China; 3 Zhengzhou Aerotropolis Institute of Artificial Intelligence, Zhengzhou 451162, China; 4 Beijing Aeronautical Technology Research Center, Beijing 100076, China

**Keywords:** machine learning, support vector regression, gray wolf algorithm, spatial mode, phase space reconstruction, wavelet singular spectrum entropy

## Abstract

The prediction of a stall precursor in an axial compressor is the basic guarantee to the stable operation of an aeroengine. How to predict and intelligently identify the instability of the system in advance is of great significance to the safety performance and active control of the aeroengine. In this paper, an aerodynamic system modeling method combination with the wavelet transform and gray wolf algorithm optimized support vector regression (WT-GWO-SVR) is proposed, which breaks through the fusion technology based on the feature correlation of chaotic data. Because of the chaotic characteristic represented by the sequence, the correlation-correlation (C-C) algorithm is adopted to reconstruct the phase space of the spatial modal. On the premise of finding out the local law of the dynamic system variety, the machine learning method is applied to model the reconstructed low-frequency components and high-frequency components, respectively. As the key part, the parameters of the SVR model are optimized by the gray wolf optimization algorithm (GWO) from the biological view inspired by the predatory behavior of gray wolves. In the definition of the hunting behaviors of gray wolves by mathematical equations, it is superior to algorithms such as differential evolution and particle swarm optimization. In order to further improve the prediction accuracy of the model, the multi-resolution and equivalent frequency distribution of the wavelet transform (WT) are used to train support vector regression. It is shown that the proposed WT-GWO-SVR hybrid model has a better prediction accuracy and reliability with the wavelet reconstruction coefficients as the inputs. In order to effectively identify the sign of the instability in the modeling system, a wavelet singular information entropy algorithm is proposed to detect the stall inception. By using the three sigma criteria as the identification strategy, the instability early warning can be given about 102r in advance, which is helpful for the active control.

## 1. Introduction

The normal operation of a compressor is directly related to the reliability and safety of the aeroengine. The instability of aerodynamic systems such as a rotating stall and surge has always been considered as one of the most difficult problems restricting the stability of the compressor. Therefore, the modeling and early detection on the rotating stall is of great significance to the aerodynamic stability of the operating system. Epstein [1] firstly proposed the concept of active control of the surge and rotating stall, which was a major breakthrough for the study of the aerodynamic instability of the compressor. Before entering into the rotating stall, there are two types of stall precursors [2,3]: small-scale spikes and large-scale modal wave. Once the stall occurs, it would not only reduce the performance of the engine, but also cause the limitation of the working range, even resulting in the damage of the whole engine. With the demand for aerodynamic performance, the problem of aerodynamic stability becomes more and more important.

Because of the limitation on the stable operation of the whole compressor, several efforts have been devised by scholars on how to detect the stall precursor as soon as possible. The methods used commonly for the detection include wavelet analysis, correlation analysis, variance analysis, harmonic Fourier transform, and so on. Chritensen [4] proposed a discrete detection method based on autocorrelation analysis and developed a control system for rotating the stall of the compressor. Liu [5] used the method of variance analysis to detect the aerodynamic instability of the compressor. Based on the mechanism of stall inception under distortion conditions, a wavelet method was adopted by Salunkhe [6] to identify the stall precursor, which could be detected 200r in advance. The method of fast wavelet was used to predict stall forewarning for the spike and modal wave patterns [7,8], but it is mainly considered to be the time information of flow by correlation analysis and variance analysis, thus ignoring the related information with the frequency domain. While taking into account the advantage of time-frequency analysis, the objective information cannot be obtained from the direct filtering data provided by wavelet analysis. How to effectively and accurately capture the stall precursor signal is still a challenge in the process of stall identification in the compressor.

Entropy as an effective data information expression has been used for the signal measurement of the complexity and uncertainty in the system. Because of the denoize effect by singular value decomposition (SVD), a hybrid approach [9] was presented to characterize the operating state in the system effectively with a combination of singular spectrum and information entropy. With proposing the WT and SVD to reflect the internal information, it provided a technique for the classification of transient signals [10]. A wavelet singular entropy algorithm incorporated with information entropy [11] was proposed to describe the distribution complexity of the spatial modalities in a dynamic system of the flow field. It was proven that, based on the principle of information entropy algorithm, the nonlinear characteristic of the dynamic system of the compressor could be well described.

The triggering mechanism of the stall inception is a complex system process, especially for the spike pattern. With establishing a connection between the nonlinear behaviors and system status parameters, the prediction on the occurrence of the rotating stall in the compressor has been carried out in practical application. An aerodynamic modeling for an aircraft stall was proposed by Gan [12] with the wavelet neural network (WNN). The results showed that this method could improve the modeling ability of the WNN, which was suitable for the aerodynamic modeling of the stall flow. Lin [13] used the radial basis function (RBF) neural network with deterministic learning to predict the bifurcation of the stall in the axial compressor under distortion conditions. By the stall data training with Long Short Term Memory (LSTM), the regression model was adopted in the neural network of the compressor [14]. The rotating stall of the axial flow compressor was predicted based on the K-means-GD-RBF fusion model [15] reconstructed from the phase space of the chaotic sequence. The global sample entropy was employed to detect the stall precursor in advance according to the difference quotient discrimination strategy.

Although these methods have made some achievements in the modeling of aerodynamic stall, the relevant modeling research is still relatively not enough. As one of the difficulties in the process of solutions on the neural network, it is easy to fall into the local minimum, resulting in difficulties on the determination of the network structure and the generalization ability. Support vector regression model has a good generalization ability in dealing with nonlinear regression problems, avoiding the minimum training, and its computation complexity does not depend on the dimension of input space. For the complex mapping relationship between the nonlinear behaviors in the flow instability, this algorithm is expected to build a closed-loop system of data and mechanism. 

As the continuation of previous research [15], the main purpose of this paper is to seek a more reliable method to model the overall system of the compressor, especially for the short-scale vortex identification from the frequency domain perspective by machine learning. The concerns to be solved include the time-space resolved diagnosis for the parameters with small-scale and multi-frequencies, as well as the optimization of the network structure parameters. Regarded as a complex dynamic system, the instability is observed to present a marked nonlinearity and chaos characteristic for the stall inception because of the unsteady sophisticated internal flow. With the data processing of parameter optimization by the GWO algorithm, an intelligent mathematical machine learning model with WT-GWO-SVR is established for the identification on the stability of the dynamic system in the flow field. Utilizing the multi-resolution and equivalent frequency distribution ability of the WT, the chaotic characteristics of the spatial mode sequence of the flow field can be mined in different frequency bands through the phase space reconstruction. In order to verify the effectiveness of the proposed model by the WT-GWO-SVR algorithm, an experimental verification was carried out on an axial flow compressor and compared with the SVR models associated with differential optimization and particle swarm optimization. The aim of this proposed hybrid model is to provide a point of view for the early intelligent identification on rotating stall inception in axial compressors with the assistance of the entropy logic solution.

Confronting the complex input-output mapping relationship, the WT-GWO-SVR algorithm is introduced for association rule learning on the flow aerodynamic parameters and instability index. The organization of this paper can be described as follows. Firstly, the introduction on the data acquisition process is represented during the rotating stall experiment. Based on the chaotic characteristics revealed, the phase space reconstruction of the spatial mode is completed by the C-C algorithm after the decomposition of the spatial mode solved by wavelet analysis. Next, for the reconstructed signal components, the SVR model is conducted for prediction on the flow system of the compressor. With the integration of the WT reconfiguration algorithm and GWO optimization algorithm, the network parameters in the SVR model can be apparently optimized, with the accuracy of the prediction improved. Finally, the wavelet singular spectral entropy analysis is carried out on the detection of stall inception according to the established WT-GWO-SVR hybrid model. The omen position of the system instability could be marked in recognition of 102r in advance, which effectively solved the criterion of the identification strategy.

## 2. Data Acquisition and Phase Space Processing

### 2.1. Introduction to Experimental Data Collection

**Figure 1:**
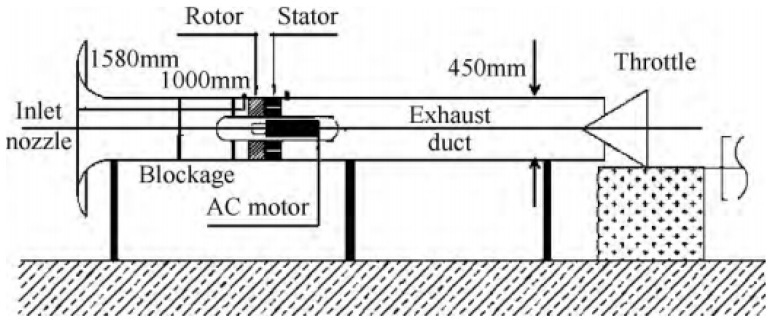
Test rig of the compressor.

The research object of this paper is a low-speed axial compressor, which is shown in the Figure 1. It consists of 19 rotor blades and 13 stator blades in a single stage at the design speed of 3000 rpm. In the process of experiment, the volume flow at the design point is 2.4 m^3^/s, with a total pressure rise of 1500 Pa. The relevant geometric parameters of the compressor are presented in Table 1 for a brief synopsis. An introduction on the test rig is described in the references [15]. 

Five Kulite dynamic pressure sensors are uniformly located in the circumference on the casing to measure the dynamic pressure. In order to be conscious of the stall inception with the variation of the pressure sensitively, the acquisition receivers of the pressure signal are placed in front of the leading edge of the rotor tip. With establishing the data acquisition system of the compressor, the pressure is recorded during the process of the stall revolution at the different working states with a sampling frequency of 5000 Hz. Because of the electromagnetic interference and acoustics in the field, an amount of noise information is included in the recorded experiment data. The operation of the noise reduction is carried out by low-pass filtering. Since the instability in the field is low-frequency information, the low-pass filtering is selected to be 200 Hz as the cut-off frequency. In order to extract the feature contained in the nonlinear data, a data fusion is processed to achieve the traveling wave by discrete Fourier transform (DFT). Then, the evolution of the modal wave in the compressor system can reveal the occurrence procedure of the stall from the perspective of modal wave energy, which lays a foundation for the establishment of modeling for the compressor system.

### 2.2. Evolution of the Spatial Mode in Flow Field

In this part, the spatial mode of the flow field is extracted from the recorded pressure signals. From the perspective of the system modal wave energy, the spatial mode in the form of traveling waves is analyzed in the time-frequency diagram, which reflects the development of the stall initiation. It provides a prerequisite for the identification of the rotating stall precursors [11]. According to the theory of spatial mode [16], the traveling stall cells can be expanded into a superposition of multiple harmonics during rotating instability. The spatial modal mathematical expression of the compressor system is defined as Equation (1),

(1) $$a_{m}=\frac{1}{K}\sum_{j=1}^{N}\Phi_{j}e^{-\frac{i2\pi mj}{N}}$$

where *a_m_* denotes the *m*-order spatial mode, *K* is the number of Kulite dynamic pressure sensors (*K* = 5), and *Φ_j_* presents the flow coefficient with the signal of the *j*-th dynamic pressure sensor.

**Figure 2:**
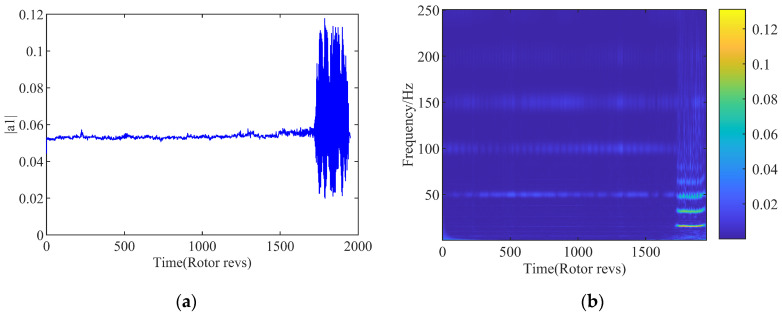
First-order spatial mode. (**a**) Time-domain diagram; (**b**) Time-frequency diagram.

It is concluded from the previous research [15] that the nonlinearity of the flow is extremely prominent at the design speed of 3000 rpm. In order to bring the superiority of the SVR machine learning model into line with the modeling nonlinear flow behavior, the main attention in this paper is focused on the variation of the first-order spatial mode at design speed. In order to speed up the efficiency on input and output calculation, the resampling and noise reduction are carried out for the spatial mode obtained in the flow field. The amplitude of the first-order spatial mode is shown in Figure 2a in the time domain. It can be seen that as the compressor system approaches the instability boundary, fluctuations begin to appear in the flow field. 

The blockage of the tip region caused by the leakage vortex movement is considered to be a major cause for the stall inception process. The formation of the large-scale stall cell is then triggered by the flow disturbance with the blocking effect. The rotational stall is an asymmetric flow along the circumference of the compressor. The amplitude of the modal energy is observed to increase obviously, corresponding to an appearance of the stall cells traveling along the circumferential direction of the compressor. By the solution of the WT, the time-frequency diagram of the first-order spatial mode is computed as shown in Figure 2b. The low-frequency information of 15 Hz can be clearly captured near 1732r, which is consistent with the result of time domain analysis. It means a traveling wave is propagating at the rotating direction of the compressor with the 15 Hz. Combined with the above analysis, it can be judged that the instability boundary of the compressor is recognized at 1732r. It is concluded that the key feature of the system is well reflected by the variation of the modal energy in the operation development of the compressor state. The sudden increase in the amplitude of the modal energy is implied accurately as the verification of the rotating stall in the system. From the view of the system identification, the information contained in spatial mode can be selected as the characterization parameter for the occurrence of the instability quantitatively. Therefore, the subsequent model of the compressor system will be developed based on the spatial modal modeling directly for the identification on the stall inception.

### 2.3. Phase Space Reconstruction of Spatial Mode

Phase space reconstruction is an important process on recovering the chaotic attractor to reflect the nonlinearity of the system. It can effectively avoid the randomness and subjectivity of the input sample data with fully displaying the characteristic information of the system contained in the sequence. Therefore, the operation of the phase space reconstruction on the first-order spatial mode is conducive to depict the nonlinear relationship of the dynamic system, which is helpful to adapt to the sequence analysis. The basic principle is described as follows.

With the chaotic time series set as $y\left(1\right),y\left(2\right),\cdots ,y(N)$, the phase space reconstruction vector can be obtained by

(2) $$Y\left(t\right)=\left\{y\left(t\right),y\left(t+\tau \right),\cdots ,y\left(t+\left(m-1\right)\tau \right)\right\},t=1,2,\cdots ,M$$

where *Y*(*t*) is the phase point of the reconstructed phase space, τ is the delay time, *m* is the embedding dimension, and *M* is the total number of phase points with M=N−(m−1)τ.

Due to the complex regularity of the influence on the stability of the flow field, the sequence of the spatial mode in the field presents chaotic characteristics [15]. In order to retain the information of system, the selection of the appropriate embedding dimension *m* and delay time τ is the key step for the phase space reconstruction. At present, the main methods for selecting the embedded dimension include the false nearest point method, Cao method, etc. The delay time can be determined according to the auto-correlation coefficient method and interactive information method. These methods require an amount of computation time with considering the embedding dimension and the delay time to be independent of each other. In fact, *m* and τ are interrelated and can be in coupling calculation. Kugiumtzis [17] revealed the relationship as shown in Equation (3),

(3) $$\tau_{w}=\left(m-1\right)\tau$$

where $\tau_{w}$ is the time delay window.

The correlation-correlation (C-C) algorithm is adopted here to reconstruct the phase space of the spatial mode. The correlation integral function expression of the time series [17] is defined as Equation (4),

(4) $$C\left(m,\tau ,N,r\right)=\frac{1}{M^{2}}\sum_{1\leq i\leq j\leq M}\theta \left(r-\left\|Y_{i}-Y_{j}\right\|\right)$$

where *r* is the control radius with positive, $\theta \left(s\right)$ is the Heaviside function, and $Y_{i}$, $Y_{j}$ are the phase points at positions *i* and *j*.

The first-order spatial modal sequence $a\left(1\right),a\left(2\right),\cdots ,a(N)$ is divided into *z* disjoint subsequences, and the test statistic of the *z* subsequence is in definition of Equation (5),

(5) $$S\left(M,\tau ,N,r\right)=\frac{1}{z}\sum_{j=1}^{z}\left\{C_{1}\left(M,\tau ,\frac{N}{z},r\right)-{C_{j}\left(1,\tau ,\frac{N}{z},r\right)}^{m}\right\}$$

where $C_{j}$ is the correlation integral of *j*-th subsequence.

When N→∞, the above definition becomes

(6) $$S\left(M,\tau ,r\right)=\frac{1}{z}\sum_{j=1}^{z}\left\{C_{1}\left(M,\tau ,r\right)-{C_{j}\left(1,\tau ,r\right)}^{m}\right\}$$

For arbitrary value of *r*, S(m,τ,r) is theoretically always to be 0, but due to the correlation between the time series, the obtained S(m,τ,r) is usually not to be 0 exactly. Therefore, the range of S(m,τ,r) is taken as the smallest time point with the difference between any radius *r.* It means that the trajectory of the chaotic attractor is fully expanded in the reconstructed phase space. The maximum deviation corresponding to the computed radius *r* is defined as follows:

(7) $$∆S\left(m,\tau \right)=max\left\{S\left(m,\tau ,N,r_{i}\right)\right\}-min\left\{S\left(m,\tau ,N,r_{j}\right)\right\},$$

According to the statistical method, the embedding dimension *m* usually ranges from 2 to 5. and $r_{i}$ values as $\frac{1}{2}\alpha i$, where α represents the mean square error of the time series. Then the equations lead to

(8) $$∆\overset{-}{S}\left(z\right)=\frac{1}{4}\sum_{m=2}^{5}∆S\left(m,\tau \right),$$

(9) $$\overset{-}{S}\left(z\right)=\frac{1}{16}\sum_{m=2}^{5}\sum_{j=2}^{4}S\left(m,\tau ,N,r_{j}\right),$$

(10) $$S_{cor}\left(z\right)=∆\overset{-}{S}\left(z\right)+\left|\overset{-}{S}\left(z\right)\right|.$$

**Figure 3:**
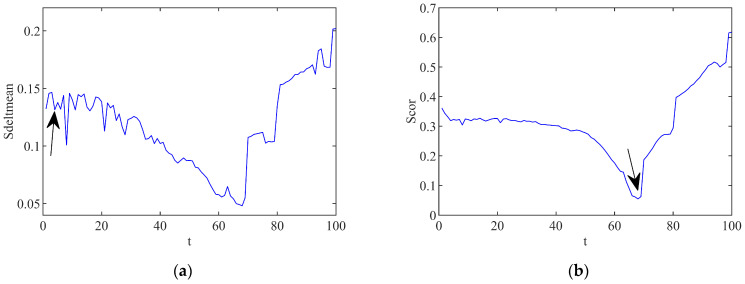
Values of $∆\overset{-}{S}\left(z\right)$ and $S_{cor}(z)$ calculated by C-C algorithm. (**a**) Curve of $∆\overset{-}{S}\left(z\right)$; (**b**) Curve of $S_{cor}\left(z\right)$.

In these formulas, the time point corresponding to the first minimum value obtained from $∆\overset{-}{S}\left(z\right)$ is the delay time τ, and the time point corresponding to the minimum value of $S_{cor}(z)$ is the time delay window $\tau_{w}$. By using the C-C algorithm, the calculation curves of the $∆\overset{-}{S}\left(z\right)$ and $S_{cor}(z)$ in the first-order spatial mode are indicated in Figure 3. According to the value definitions of the formulas, the value points are indicated by the arrows in Figure 3a,b. 

It can be analyzed from Figure 3 that the delay time τ of the spatial mode reconstruction is valued as 4 with the time delay window $\tau_{w}$ to be 68. According to the relationship between the embedding dimension and the delay time given by Equation (3), the embedding dimension *m* is calculated to be 18. Through the phase space reconstruction, the internal information of the flow space mode can be displayed more clearly. Next, the modeling of the aerodynamic system will be conducted based on the phase space reconstruction of the space mode in the coming chapter of this paper.

## 3. Modeling Methodology with Support Vector Regression

### 3.1. Basic Theory of Support Vector Regression Algorithm

Support vector regression (SVR) is a method derived from support vector machine (SVM) [18], which is widely used in nonlinear regression problems and system identification modeling. The main logic of SVR is to construct a linear decision function of a high-dimensional feature space instead of the nonlinear function of the original space by using a nonlinear mapping for the sample data. Hence, the computational complexity of SVR does not depend on the dimension of the input space. While solving the dimensional problem, it also ensures a good ability of generalization. Therefore, taking advantage of the excellent generalization ability of SVR, the model of the spatial mode in the system is to be established in this paper.

Assume the training sample set to be

(11) $$\left\{\left(x_{j},y_{j}\right)|x_{j}\in X=R^{d},y_{j}\in Y=R,j=1,2,\cdots ,n\right\},$$

where $x_{j}$ is the input value and $y_{j}$ is the output value. The nonlinear mapping φ(.) is adopted to map the input space data into the high-dimensional feature space, and then construct a function in the feature space that is highly approximate to the training sample, defined as,

(12) $$f\left(x\right)=w\cdot \varphi \left(x\right)+b,$$

where *w* is the weight coefficient vector, *b* is the bias vector.

Considering the allowable regression fitting error, the relaxation factors $\zeta_{j}$ and $\zeta_{j}^{*}$ are introduced. Accordingly, each sample in the training sample set satisfies the following constraints,

(13) $$\left\{\begin{array}{c} y_{j}-\left(w\cdot \varphi \left(x_{j}\right)\right)-b\leq \varepsilon +\zeta_{j},j=1,2,\cdots ,n\\ \left(w\cdot \varphi \left(x_{j}\right)+b\right)-y_{j}\leq \varepsilon +\zeta_{j}^{*}\\ \zeta_{j}\geq 0,\zeta_{j}^{*}\geq 0 \end{array}\right.,$$

The optimization objective is set as [19],

(14) $$\begin{array}{c} \underset{w,b,\zeta_{j},\zeta_{j}^{*}}{\min}\left\{\frac{1}{2}{\left\|w\right\|}^{2}+C\sum_{j=1}^{n}\left(\zeta_{j}+\zeta_{j}^{*}\right)\right\}, \end{array}$$

where *C* is the penalty factor with positive.

In order to optimize the target, the convex quadratic programming problem of Equations (13) and (14) is solved by the dual theory and constructed the Lagrangian function. The obtained function is shown as,

(15) $$\begin{array}{c} f\left(x\right)=\sum_{j=1}^{n}\left(a_{j}^{*}-a_{j}\right)\cdot K\left(x_{j},x\right)+b, \end{array}$$

where the Lagrange multipliers are expressed as $a_{j}^{*}$, $a_{j}$, the kernel function indicates as $K\left(x_{j},x\right)=e^{\frac{-{\left\|x_{j}-x\right\|}^{2}}{2g^{2}}}$.

Based on the above analysis, it can be concluded that the accurate selections of the model parameters, namely as penalty factor *C* and kernel function parameter *g*, are the key to solve the problem of regression prediction by using the support vector machine. The setting of parameters has a great impact on the prediction accuracy of the SVR model. The generalization ability of the model is reflected by the smoothness of the regression curve and the compromise of the empirical risk, which are controlled by penalty factor *C*. The kernel function is the key factor to determine the nonlinear mapping ability of the model, and a similar accuracy of prediction can often be obtained by different types of kernel functions. That means the support vector machine does not depend on the type of kernel function to a certain extent, but the parameter *g* of the kernel function has a great influence on the performance of SVR model. Therefore, in this paper, the parameter g of the RBF kernel function is chosen here to study the performance of SVR. However, in the past, the determinations of the parameters in the modeling with SVR are mostly manual [20]. The computation cost would be in a large amount with a wide range searching for parameters manually. In view of these shortcomings, the gray wolf optimization algorithm is adopted in this paper for the selection of the optimal parameters.

### 3.2. Support Vector Regression Prediction Model Based on Gray Wolf Optimization Algorithm

With the development of metaheuristic algorithms in recent years, more accurate optimization solutions have been handled for complex engineering problems. Nadimi [21] proposed an improved gray wolf optimizer (I-GWO) to solve global optimization and engineering design problems. The experimental results showed that the I-GWO algorithm had a strong competitiveness and applicability. A multi-trial vector-based differential evolution algorithm (MTDE) [22] was proposed to solve the complex optimization problems. The algorithm indicated a high precision of optimal solution for engineering design problems. By maintaining specific behaviors during the optimization process, the multi-trial vector-based moth-flame optimization algorithm (MTV-MFO) was proposed with different types of multi-trial vector generators [23]. The results showed that the MTV-MFO algorithm had a great advantage in accuracy and convergence speed. An improved whale optimization algorithm (EWOA) [24] was developed for the optimal power flow problem (OPF). This algorithm can improve the exploration ability and maintain an appropriate balance between the exploration and development. According to the applicability of the optimization algorithms, this paper would focus on the application of the GWO algorithm because of the global optimization for the engineering problem.

The gray wolf optimization algorithm (GWO) is a developed swarm intelligence optimization algorithm inspired by the predatory behavior of gray wolves [25]. In terms of convergence speed and global search performance, this GWO algorithm is superior to algorithms such as differential evolution and particle swarm optimization, and it is widely used in technical fields such as parameter optimization [26]. In the population of gray wolves, there is a strict social dominance hierarchy.

The hierarchy of gray wolves ranks from high to low as α, β, δ, and ω. The hunting behaviors of gray wolves are mainly dependent by wolves α, β, and δ. The optimization process of GWO is primarily guided by the optimal solution α and suboptimal solution β, δ in each generation of population. The predation behavior of gray wolves is defined as

(16) $$D=\left|G\cdot X_{q}\left(t\right)-X\left(t\right)\right|,$$

(17) $$X\left(t+1\right)=X_{q}\left(t\right)-A\cdot D,$$

(18) $$A=2a\cdot r_{1}-a,$$

(19) $$G=2r_{2},$$

where *t* is the current iteration number, *D* is the distance between gray wolves and their prey, *A* and *G* are synergy coefficient vectors, $X_{q}\left(t\right)$ is the position vector of prey in the *t*-th iteration, $X\left(t\right)$ is the position vector of the wolf in the *t*-th iteration, namely as the current local optimal position. The modulo values of $r_{1}$ and $r_{2}$ are uniformly distributed random numbers between [0, 1], and *a* is the convergence factor. 

A mathematical model of the wolves behavior in tracking prey is sketched as

(20) $$D_{\alpha }=\left|G_{1}\cdot X_{\alpha }-X\right|,$$

(21) $$D_{\beta }=\left|G_{2}\cdot X_{\beta }-X\right|,$$

(22) $$D_{\delta }=\left|G_{3}\cdot X_{\delta }-X\right|,$$

where $G_{i}(i=1,2,3)$ is the random vector, *X* is the current position of the gray wolf. $X_{\alpha }$, $X_{\beta }$ and $X_{\delta }$ are the current optimal solution positions of the α, β, and δ wolves, and $D_{\alpha }$, $D_{\beta }$ and $D_{\delta }$ are the distance between the α, β, and δ wolves and other individuals. Thus, the optimal position of the gray wolf is determined by

(23) $$\begin{array}{c} X\left(t+1\right)={\frac{1}{3}(X}_{1}+X_{2}+X_{3}), \end{array}$$

where $X_{1}=X_{\alpha }-A_{1}\cdot D_{\alpha }$, $X_{2}=X_{\beta }-A_{2}\cdot D_{\beta }$, $X_{3}=X_{\delta }-A_{3}\cdot D_{\delta }$ with the random vector $A_{i}(i=1,2,3)$.

### 3.3. Gray Wolf Algorithm Optimized Support Vector Regression Model Combined with Wavelet Transform

**Figure 4:**
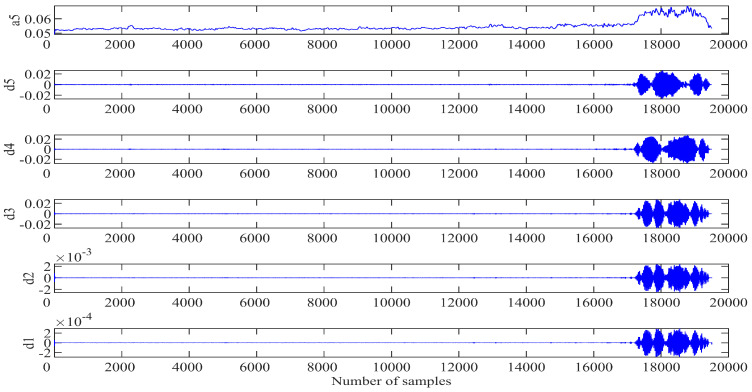
Reconstruction coefficient of wavelet multiscale decomposition.

After the engine system entering into instability, the information of the unsteady flow presents the characteristics of multi-category, multi-parameter, and multi-dimensional random data. Therefore, the procedure of the system instability is a complex nonlinear dynamic system evolution process. Since the SVR model cannot eliminate the defect of prediction error caused by uncertain factors, the wavelet transform (WT) provides a tool to improve the prediction accuracy of the dynamic system instability. Based on the GWO-SVR modeling progress, a hybrid prediction model is proposed in this part in combination with the wavelet transform, named as the WT-GWO-SVR model. Using the multi-scale analysis of the wavelet transform to preprocess the space mode obtained, the db5 wavelet basis function is selected to decompose and reconstruct the first-order space mode at 5 scales. Since the information of the original spatial mode will be lost when the number of decomposition levels exceeds 5, the optimal number of wavelet decomposition levels is 5, as shown in Figure 4.

**Figure 5:**
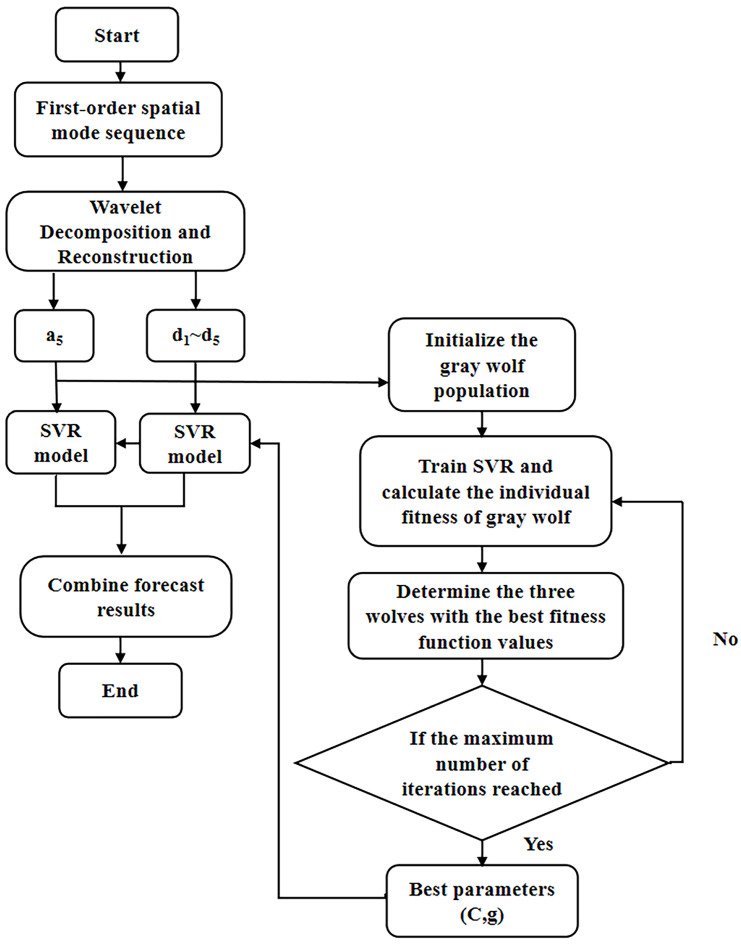
Flow chart based on WT-GWO-SVM hybrid model.

Figure 4 shows the approximate component a5 (low frequency) of the fifth layer and the other detail components d1 to d5 (high frequency) of each layer. The approximate component represents the change trend of the first-order spatial mode, accounting for the majority of the whole information. The phase space of each component is reconstructed and divided into two parts: the training sample set and the test sample set. Then the GWO optimization algorithm is applied to determine the penalty factor C and the kernel parameter g of the radial basis function. The population size of the wolves is set to be 20 with the maximum number of iterations as 100. The system modeling with SVR is trained according to the normalized training sample data and the individual fitness function values of the gray wolf calculated. The position information of the three wolves with the best fitness function value is saved for next step. The location of the individual gray wolf can be constantly updated through the adaptive iterative program with the best fitness value as the output for the optimal parameter combination (C, g). The SVR model with optimized parameters is checked with the test sample data for evaluation the final generalization ability of the SVR prediction model, and the prediction of each component is obtained through the inverse normalization processing. Finally, the results of the approximate components and detail components are combined together to achieve the final prediction of the spatial mode in the field [27]. The modeling process of WT-GWO-SVR hybrid model is indicated in Figure 5.

The mean absolute error (MAE), root mean square error (RMSE), and R square ($R^{2}$) are introduced to evaluate the performance of the SVR model. The MAE reflects the actual error of the predicted value. The deviation between the predicted value and the actual value is measured with the RMSE. The fitting degree of the prediction is reflected by $R^{2}$ with a better accuracy as the value to be close to 1. The specific definitions are expressed as

(24) $$MAE=\frac{1}{n}\sum_{j=1}^{n}\left|y_{j}-\tilde{y_{j}}\right|,$$

(25) $$RMSE=\sqrt{\frac{1}{n}\sum_{j=1}^{n}{\left(y_{j}-\tilde{y_{j}}\right)}^{2}},$$

(26) $$R^{2}=1-\frac{\sum_{j=1}^{n}{\left(y_{j}-\tilde{y_{j}}\right)}^{2}}{\sum_{j=1}^{n}{\left(\overset{-}{y}-y_{j}\right)}^{2}},$$

where $y_{j}$ is the actual value of the *j*-th sample, $\tilde{y_{j}}$ is the predicted value of the *j*-th sample, $\overset{-}{y}$ is the average of the actual value, and *N* is the total number of samples.

**Figure 6:**
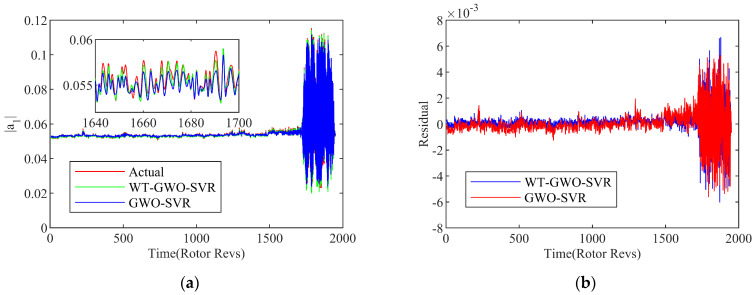
Results of WT-GWO-SVR and GWO-SVR hybrid model. (**a**) Prediction curve; (**b**) Residual curve.

With the selection of the embedding dimension *m* as 18, the phase space reconstruction of the first-order space mode is performed, and the value of each step is predicted according to the 18 steps prior to the current location. Figure 6 show the single-step prediction results of the first-order space mode on the basis of the hybrid SVR models at 3000 rpm. Although there are still errors between the actual value and the prediction, the extreme high reliability and accuracy are fully implied in the hybrid SVR modeling the system for the overall operating state. 

In order to inspect the superiority of the WT-GMO-SVR model, the prediction performance is contrasted with the GWO-SVR model. In order to measure the optimization capability on the parameters, the SVR models are built up with the same training condition for modeling the system by using the DE algorithm and PSO algorithm. With a contrastive study on the performance of the above two prediction models quantitatively, the measurements as MAE, RMSE and $R^{2}$ are adopted in this paper for comprehensive evaluation, as indicated in Table 2. All the comprehensive indexes of the WT-GWO-SVR prediction model proposed are superior to the other models. Moreover, it is indicated that the established SVR hybrid model in this research reflects a better modeling ability with an accurate prediction, as compared to the RBF hybrid neural network model. From the analysis above, it can be concluded that the hybrid network modeling method proposed in this paper has revealed an outstanding performance for the nonlinear dynamic system prediction. With the integration of the WT reconfiguration algorithm and GWO optimization algorithm, the accuracy of the prediction model with SVR can be effectively improved, as displayed by the evaluation indicator comparisons with the other methods mentioned above.

## 4. Identification Strategy on the Aerodynamic System Instability

### 4.1. Instability Identification Design with Single Step Based on Wavelet Singular Spectrum Entropy Algorithm

**Figure 7:**
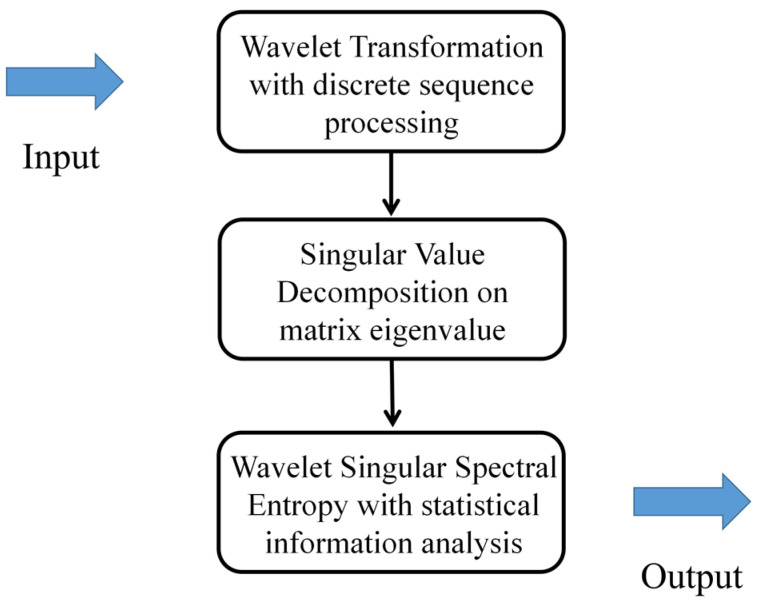
Diagram of the process with the wavelet singular spectral entropy algorithm.

When the instability of a compressor occurs, the distributions of normal signals and unstable signals are different in time-frequency space. Therefore, an effective method needs to be considered to accurately distinguish the nuance of the sequence. Aiming to achieve an available method of identification on the instability precursor, the algorithm of wavelet singular spectrum entropy (WSE) is adopted here to measure the dynamic mutation in the time-frequency series [28]. The main mentality is to transform the wavelet coefficient matrix of the denoize data into a series of probability distributions. With decomposing the sequence of distributions into a set of singular values, the basic feature can be then reflected through the singular value decomposition [29]. The uncertainty of the singular values is computed by the information entropy algorithm [7] for the discrete sequence of the system. The procedure of data processing is given a detailed description in document [11]. The diagram of the technological process based on the wavelet singular spectral entropy algorithm is drawn in Figure 7.

**Figure 8:**
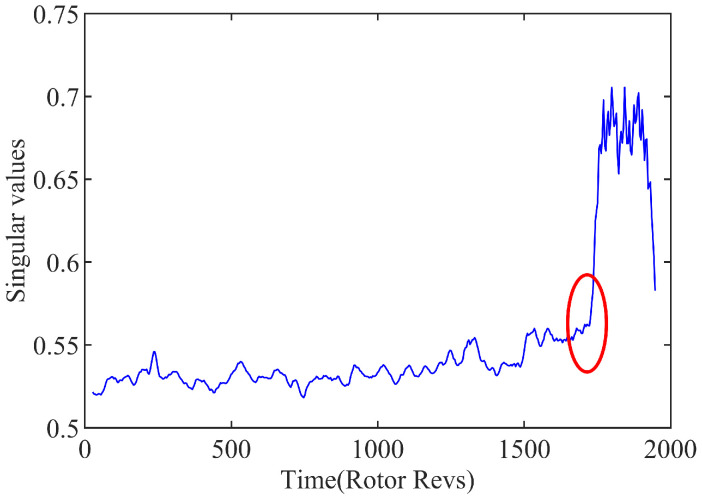
Variation of singular values in sliding windows.

By calculating the wavelet singular spectrum entropy of the spatial mode in the compressor system, the precursor of rotating the stall can be identified by using the difference of entropy before and after the instability. According to the discussion on the parameter selection of the wavelet singular spectral entropy [11], the sliding window is set as 100, and the sliding step size is set as 20 to analyze the spatial pattern predicted for the inception recognition. In order to assess the wavelet singular spectrum entropy conveniently, the singular eigenvalues are calculated for each window, as shown in Figure 8. The singular values of spatial pattern can be effectively extracted up as the indexed signal to restore the basic feature of the system. And it can be used to quantitatively distinguish the distinction cross the instability boundary in the modal space of system, as circled in the chart.

**Figure 9:**
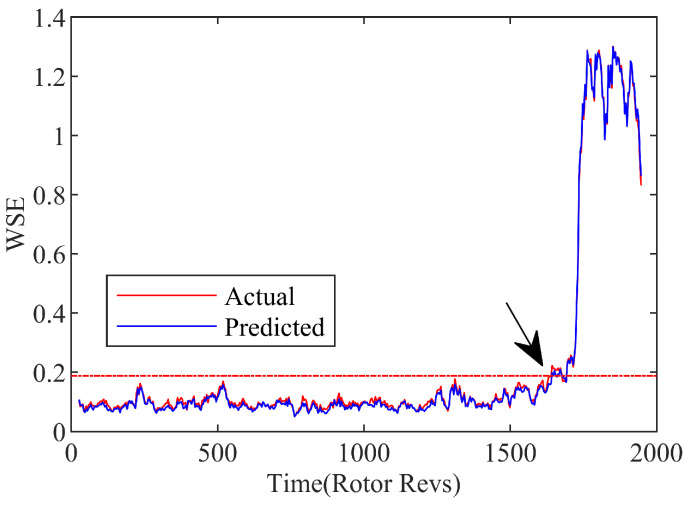
Wavelet singular spectrum entropy of spatial mode.

Considering the sensitivity and accuracy of current recognition, the contrast of wavelet singular spectrum entropy is obtained between the prediction SVR model and experiment data, as indicated in Figure 9. When the compressor approaches the stall boundary, the corresponding amplitude of the wavelet singular spectral entropy (WSE) increases significantly. This kind of appearance can be regarded as a marker of instability, which exhibited an obvious dissimilarity with the normal state. The consistency of the overall fluctuation trend and the small difference of the comparison results once again prove the accuracy of the modeling compressor system with the SVR function. It lays a reliable foundation for the prediction on the occurrence of the rotating stall precursor based on the WT-GWO-SVR model.

**Figure 10:**
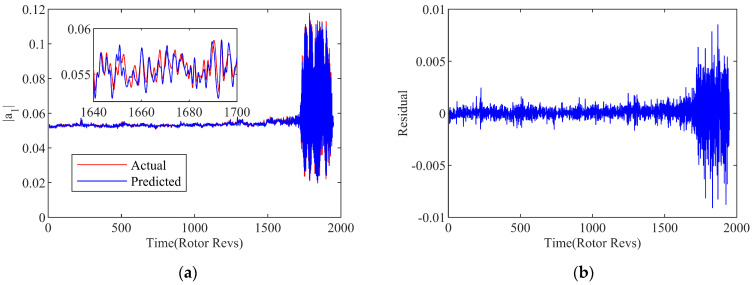
10-step prediction of WT-GWO-SVR hybrid model. (**a**) Prediction curve; (**b**) Residual curve.

In order to accurately identify the occurrence location of the stall precursor, the strategy with the recognition threshold ε is set as the mean value plus three times the standard deviation according to the 3*σ* principle. The average WSE under normal conditions is taken as the benchmark. The threshold is indicated as

(27) $$\varepsilon =\overset{-}{\omega }+3\sigma ,$$

where the standard deviation is expressed by σ, and the mean value of the WSE in normal conditions is represented by $\overset{-}{\omega }$. The location where the amplitude of the WSE exceeds the set threshold is considered as the initial stall disturbance of the compressor system. As represented by the arrow point at the intersection of the dashed threshold and the entropy signal in Figure 10, the stall inception is recognized at approximately 1637r. Therefore, based on the WT-GWO-SVR hybrid model with the single-step prediction, the stall precursor can be detected about 95r in advance by means of the identification strategy with the wavelet singular entropy algorithm.

### 4.2. Foregleam with Multi-Step Prediction by Wavelet Singular Spectrum Entropy Identification Strategy

It is of great significance for the safe operation of the aeroengine to provide an accurate prescient warning of the instability in advance. For the aim of achieving a greater time margin for early warning, a foregleam with multi-step prediction is explored in this section for the inception recognition. The property of the flow instability can be distinguished with an earlier location, which is supposed to be superior to the single-step strategy previously introduced. However, as the steps of the prediction increase, the accuracy of the prediction also decreases to a certain extent. Therefore, it is meaningful to select an appropriate number of prediction steps for the multi-step identification strategy.

**Figure 11:**
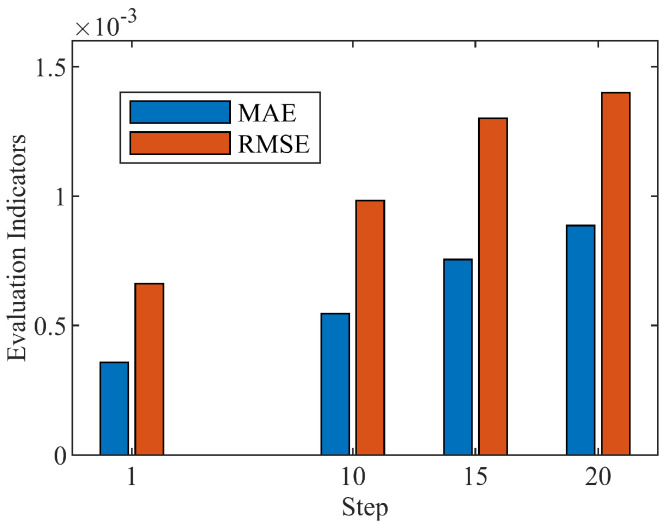
Evaluation indexes of different predicted steps.

Suppose the network input of the multi-step prediction model as $X\left(t\right)=\left\{x\left(t\right),x\left(t+\tau \right),\cdots ,x(t+(m-1)\tau )\right\}$, the corresponding network output is $Y\left(t\right)=y\left(t+h+\left(m-1\right)\tau \right)$, where t=1,2,⋯,N−(m−1)τ, and *h* is the number of prediction steps. In order to verify the accuracy of the multi-timesteps prediction model, examinations with 10/15/20 steps of prediction on the instability are carried out by the WT-GWA-SVR modal model with the phase space transformation. The exhibitions of results are drawn up in Figure 10 and Figure 11. It can be seen that the predicted trend is basically consistent with the amplitude of the spatial mode in the experiment. The residual error of the calculation result is kept within a certain low range, proving an acceptable effect achieved.

Simultaneously, as to assess the performance of the multi-step prediction method, the MAE and RMSE evaluation indexes are indicated here to compare the performances of the different prediction steps. The overall error of the prediction performance is reflected by the indicator of the MAE, while the dispersion degree of the sample data is measured by the RMSE. From the comparison shown in Figure 11, the slight larger errors appear among the multi-step prediction method in contrast with the single-step model introduced in the previous section. Yet the level of error is still controlled in a low range, which ensures the prediction accuracy of the WT-GWA-SVR model with multi-timesteps.

**Figure 12:**
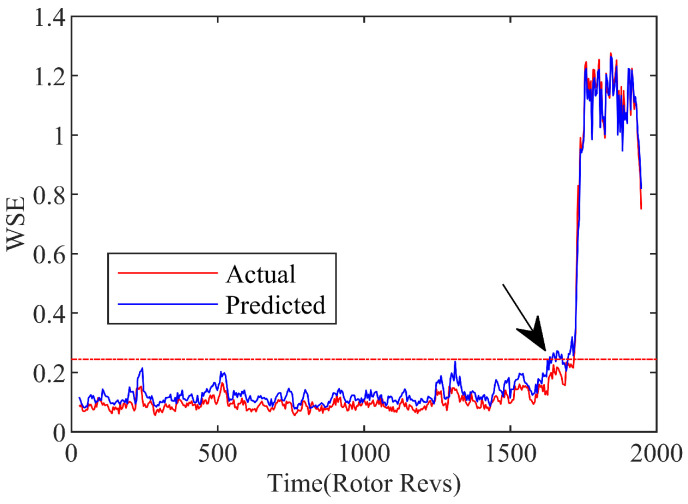
Wavelet singular spectrum entropy of spatial mode with multi-step prediction.

Figure 12 reveals the curve of the wavelet singular spectrum entropy with the 20-step prediction model, as well as the actual processing data of the experiment. Although the wavelet singular spectrum entropy predicted by the multi-step method deviates a bit from the actual value, it is still under the domination within a certain deviation tolerance range. Similarly, the stall precursor recognition threshold is set according to the 3σ principle, as expressed by Equation (27). The intersection of the dotted threshold line and entropy signal is considered to be the location of the stall precursor predicted, which is represented by arrow at approximately 1634r. Because of the 20 timesteps corresponding to 0.08 s, thus the efficient prediction point is 0.08 s (4R) ahead of the identification spot. Therefore, on account of WT-GWO-SVR hybrid multi-step prediction model, the system instability of the compressor can be in recognition of 102r in advance by the wavelet singular entropy identification strategy.

## 5. Discussion

It is very important to predict and identify the rotating instability in advance for the safe operation of the aeroengine. It contains a multi-dimensional and multi-standard patterns in the flow field data of the aerodynamic system in the compressor. Therefore, it is necessary to accurately mine the data features. On this basis, the machine learning hybrid model can be established successfully for the system status of the compressor. Therefore, in this paper, the pressure signals of the flow field are converted into the spatial modes in the form of traveling waves for the description of the flow instability. Moreover, the WT-GWO-SVR hybrid model is conducted for the prediction on the spatial mode reconstructed by single-step and multi-step modeling.

In practice, multi-step prediction may better satisfied the demand of a prescient warning at the risk of the stall than the single-step prediction. In the research of this paper, a better prediction accuracy in the single-step modeling is shown. For the multi-step prediction, the cumulative error may gradually gather in the accretion with the increase in the prediction steps. Therefore, the balance between the model accuracy and warning margin is the key point to the research of the instability identification. In the program computation, the Intel single core i5 processor is employed for the single-step prediction with 0.016 s. With a relative complex calculation process, the cost for the multi-step prediction is 0.161 s, which is one more order of magnitude than the former for the calculation time. Actually, in the multi-step calculation, more time has been spent than the timesteps itself (0.08 s) in advance.

Although the early warning of the instability is obtained with an accurate determination in this paper, the application of the SVR fusion model of the compressor system based on the machine learning algorithm yet needs a further research and verification. The accuracy of the WT-GWO-SVR hybrid model still needs an in-depth improvement, especially for the prediction with more forecast steps in advance. The other metaheuristic algorithms for improving aerodynamic modeling are worth further exploration, such as the dragonfly optimization algorithm, the whale optimization algorithm, and so on.

## 6. Conclusions

In the cross study of the flow information and machine learning modeling, a hybrid SVR model is proposed for modeling the aerodynamic system of the flow field, which breaks through the fusion technology based on the feature correlation of chaotic data. The solution of the wavelet transform is used to decompose the frequency components with the C-C algorithm for the phase space reconstruction. Optimized by the gray wolf optimization algorithm, the WT-GWO-SVR model is established for the system information modeling. Focusing on the identification strategy, the wavelet singular spectrum entropy algorithm is introduced to set the recognition threshold, so as to realize the detection of the stall precursor in the compressor. The main conclusions are summarized as follows.

(1) In the processing of the feature correlation, the spatial modes are extracted according to the pressure data of multiple measuring points. The sudden increase in the amplitude of the modal energy is implied accurately as the verification of the rotating stall in the system. From the view of the system identification, the information contained in the spatial mode can be selected as the key parameter for the occurrence of the instability quantitatively. Because of the chaotic characteristic represented by the sequence of the spatial modal, the solutions of the WT and CC algorithm are adopted to decompose and reconstruct the phase space in the field. The local signal components are obtained with different frequency information, associated with the characteristic the stall precursor.
(2) On the premise of finding out the local laws of the system, the SVR method is used to model each reconstructed frequency component. With the determination of the embedding dimension and the delay time of the sequence optimized by the GWO algorithm, the mathematical correlation between the aerodynamic parameters and the instability index is effectively built up by the WT-GWO-SVR hybrid model. Taking cluster analysis and gradient optimization as the way, the data modeling process is established based on data fusion and feature association. It is indicated that the established the SVR hybrid model in this research reflects an outstanding performance for the nonlinear dynamic system prediction. In order to achieve a longer forecast margin, the research on the multi-step prediction is continued to carry out on the hybrid modeling for the system information. It has still achieved an acceptable accuracy for the prediction with multi-timesteps.
(3) In order to effectively identify the sign of the instability in the modeling system, a information entropy algorithm is proposed to detect the stall inception. Focusing on the time-frequency analysis, the wavelet singular spectrum is introduced to describe the instability process of the dynamic system. According to the obvious difference between the entropy weights under the normal and stall conditions of the compressor, the instability boundary can be well distinguished in the modal space of system. By designing the three sigma criteria to design the recognition threshold strategy, the warning location of the stall precursor can be suggested about 102r in advance with the multi-step prediction, showing a good accuracy and application prospect.

## Figures and Tables

**Table 1:** Geometric parameters of the device.

Parameter	Value
Design speed n (rpm)	3000
Outer diameter D (mm)	450
Blade height h (mm)	56
Tip speed (m/s)	70.7
Hub-tip ratio	0.75
Rotor blade number	19
Stator blade number	13

**Table 2:** Comparison of WT-GWO-SVR model and the other models.

Model Type	MAE × 10^−4^	RMSE × 10^−4^	R^2^ × 10^−4^
WT-GWO-SVR	3.5753	6.6172	0.9923
GWO-SVR	4.8937	8.4660	0.9874
DE-SVR	7.9318	9.9174	0.9827
PSO-SVR	5.7468	8.8422	0.9863
Chaos-K-means-GD-RBF [17]	12.01	26.13	

## Data Availability

Not applicable.
